# Photons detected in the active nerve by photographic technique

**DOI:** 10.1038/s41598-021-82622-5

**Published:** 2021-02-04

**Authors:** Andrea Zangari, Davide Micheli, Roberta Galeazzi, Antonio Tozzi, Vittoria Balzano, Gabriella Bellavia, Maria Emiliana Caristo

**Affiliations:** 1 Pediatric Surgery and Urology Unit, Azienda Ospedaliera San Camillo Forlanini, Circonvallazione Gianicolense 87, 00152 Rome, Italy; 2 Wireless Access Engineering Department, TIM S.P.A., Via Oriolo Romano, 240, 00189 Rome, Italy; 3 Departement of Life and Environmental Science, Università Politecnica delle Marche, via Brecce Bianche, 60131 Ancona, Italy; 4 UOC Fisica Sanitaria, Azienda USL Toscana Sud Est, via Senese 161, 58100 Grosseto, Italy; 5 UOC Anatomy and Pathological Histology, Azienda Ospedaliera San Camillo Forlanini, Circonvallazione Gianicolense 87, 00152 Rome, Italy; 6 Explora Biotech Srl, via G. Peroni 386, 00131 Rome, Italy; 7 Centro Ricerche Sperimentali, Università Cattolica del Sacro Cuore, Largo Agostino Gemelli, 1, 00168 Rome, Italy

**Keywords:** Biochemistry, Neuroscience, Neuronal physiology, Action potential generation

## Abstract

The nervous system is one of the most complex expressions of biological evolution. Its high performance mostly relies on the basic principle of the action potential, a sequential activation of local ionic currents along the neural fiber. The implications of this essentially electrical phenomenon subsequently emerged in a more comprehensive electromagnetic perspective of neurotransmission. Several studies focused on the possible role of photons in neural communication and provided evidence of the transfer of photons through myelinated axons. A hypothesis is that myelin sheath would behave as an optical waveguide, although the source of photons is controversial. In a previous work, we proposed a model describing how photons would arise at the node of Ranvier. In this study we experimentally detected photons in the node of Ranvier by Ag^+^ photoreduction measurement technique, during electrically induced nerve activity. Our results suggest that in association to the action potential a photonic radiation takes place in the node.

## Introduction

Neural communication represents one of the most complex functions of biological systems. Signaling between cells and organs, either by proximity or across a distance, is essentially accomplished through a multitude of biochemical mechanisms. Although such communication mechanisms are ubiquitous in cellular systems, including nervous system, what trivially characterizes nervous cell is that its function is communication itself. Key features of neurotransmission are speed, synchrony and the amount of information transfer. An explanation of such properties required to consider a form of energy transfer emerging from, but not limited to, molecular contact and interaction. The fundamental theory formulated by Hodgkin and Huxley explains the basic mechanism of neurotransmission by a sequential activation of local ionic currents^1^. Since what are usually referred to as electrophysiological phenomena are ultimately electromagnetic in nature, this theory not only provides a sound explanation of an important physiological mechanism, but also opens up the possibility of providing new contributions to neuroscience by resorting to more comprehensive electromagnetic approaches. In the last decades research gradually began to deal with the implications of electromagnetic fields generated by brain activity, both as a whole and at the level of individual cells^2–7^. In particular, electromagnetic waves in the optic and infrared range, have been attracting growing attention for their possible role in neural communication^8–10^. The presence of photons during neural activity was experimentally demonstrated by optical detection^11^ and more recently by a technique of in situ biophoton autography, based on the principle that ionic Ag^+^ in solution precipitates as insoluble Ag granules when exposed to light^12^. Recent studies have put forward possible explanations of such experimental evidence. Kumar et al. have speculated that myelinated axon may behave like an optical waveguide^13,14^. Liu et al. reported myelin sheath as a way for dramatic speed enhancement of signal propagation in nerves in the THz-infrared range^15^. Jingjing Xu et al. mimicked the myelin sheath structure in myelinated axons^16^. They showed a clear confinement effect for the energy flux of transmitting electromagnetic waves inside a dielectric tube, strongly supporting the model of soft material waveguide. Zuoxian Xiang, et al., reported a primary model of THz and far-infrared signal generation and conduction in neuron systems^17^. Song et al. reported relatively strong coupling of the mid-infrared photon with the vibrons of phospholipid tails in the myelin. They proposed that cell vibron polariton in myelin sheaths may provide a promising way for super-efficient consumption of extra-weak bioenergy and even directly serve for quantum information^18^.

However, the question of the source of the radiation arose. Recently our group described a model in which electromagnetic waves—in the infrared and optical wavelength range—would be generated at the node of Ranvier, where the sodium currents would behave as an array of emitting nanoantennas^19^. Although the presence of photons has been experimentally proven in active nerve tissues, their occurrence in particular cell compartments, such as the nodes of Ranvier, has not yet been studied.

Aim of this study is to investigate the possibility that light-induced reduction of silver occurs in the node of Ranvier, during electrical stimulation of a peripheral nerve.

## Results

### Silver deposits in the nodes of Ranvier

**Figure 1:**
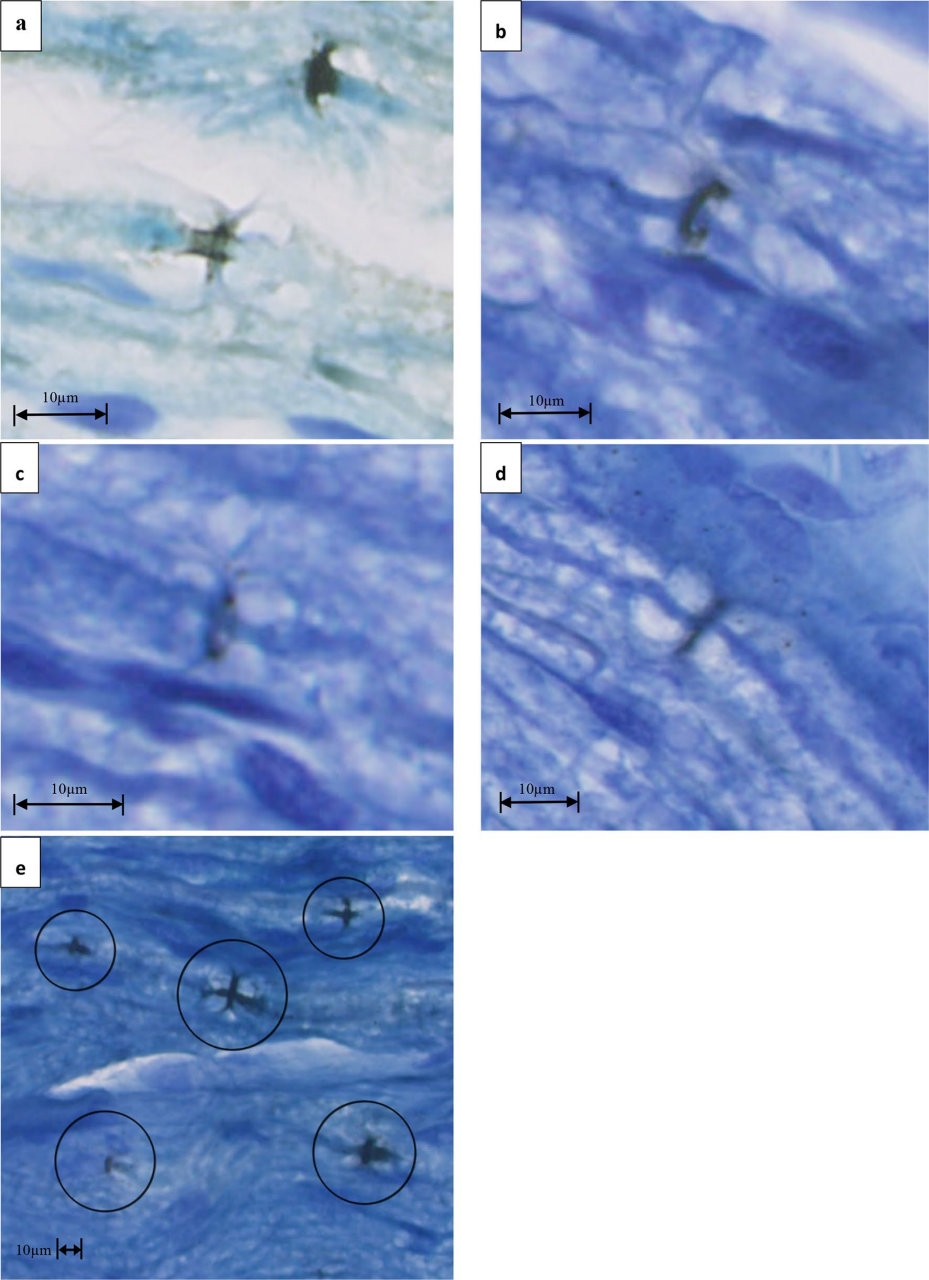
Examples of nodes where Ag deposited after electrical stimulation of rat sciatic nerves. In (**a**–**d**) a zoom of some annular Ag depositions in the nodes are displayed. Circles in (**e**) put in evidence Ag deposition in the nodes and along paranodal segments.

In order to investigate bio-photonic activity in nervous tissue, Sun et al. developed a new method called in situ biophoton autography (IBA), which is described in detail in the methods section^12^. This method relies on Ag^+^ to Ag reduction by means of exposure to light as in photography; Ag deposition in the tissue, after stimulation in a dark environment, can be identified under a light microscope, thus tracing the occurrence of photons. In their study, nervous tissue stimulation was obtained by light. By modifying IBA, we have developed a method to detect photons in the node of Ranvier associated to electrical stimulation of nerves. Likewise, the experimental set up was designed to include the sample in a sealed dark container, in order to ensure total insulation from external light during stimulation. This is explained in detail in the methods section. However, we used electrical stimulation instead of light to elicit action potentials, so as to avoid the presence of any photons coming from external light sources and thus obtaining evidence that photons emerge from cellular processes and are not introduced as such. Silver deposits in the nodes of Ranvier are visible as black or dark gray granules in a blue background, obtained using a standard blue toluidine staining technique, which makes nodes and other axonal structures clearly recognizable. Examples of nodes where Ag deposited after electrical stimulation of rat sciatic nerves are shown in Fig. 1.

**Figure 2:**
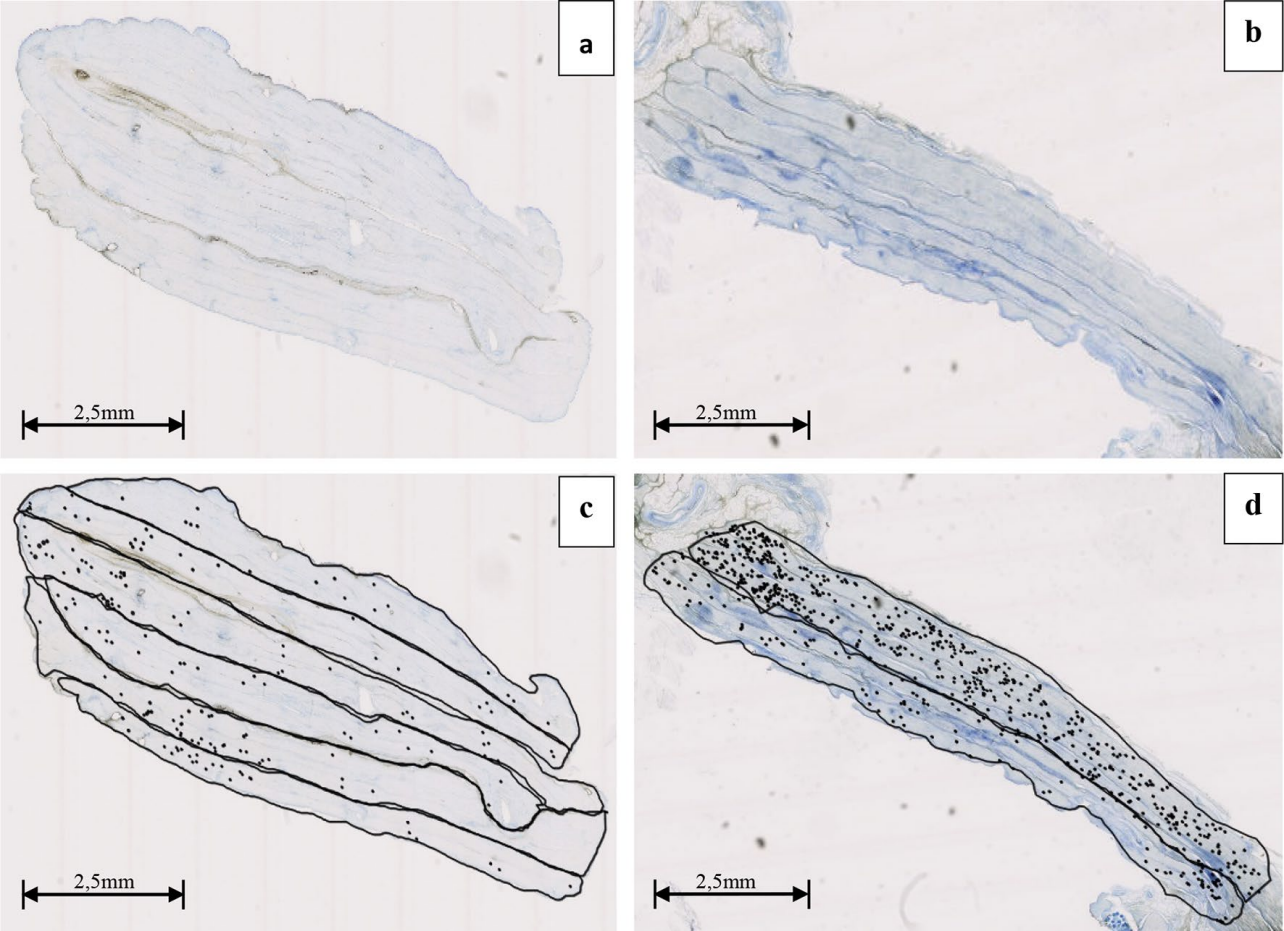
Examples of rat sciatic nerve sections (**a**,**b**). Node count, by small circle markings, in delimited fascicular areas in sections from non-stimulated nerve (**c**) and stimulated nerve (**d**).

Six sciatic nerves were harvested from Wistar rats. Stimulation was applied to 6 nerves, whereas 8 nerve segments were used as controls. Sections for histology where obtained from the specimens: 10 from the stimulated segments and 9 from the controls (Fig. 2a, b). Small sample areas, corresponding to nerve fascicles, were identified and marked in all sections, for a total number of 50 in stimulated group and 43 in controls (Fig. 2c, d).

**Figure 3:**
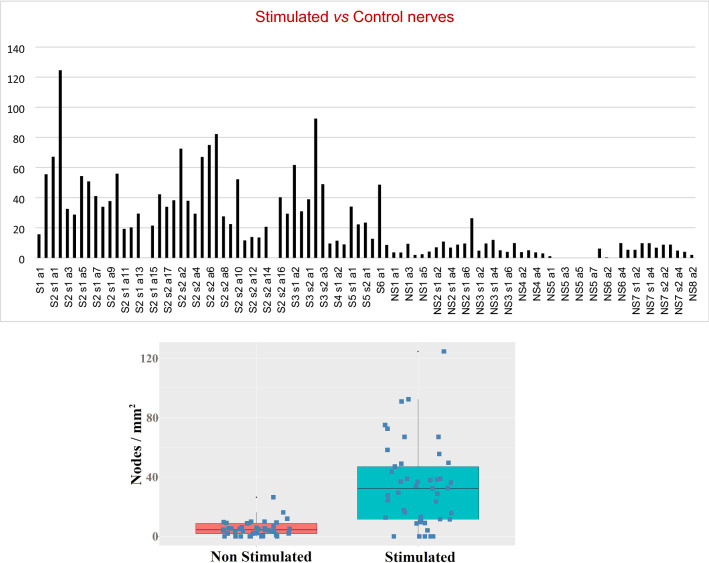
**Up:** Nodes’ density in sample areas in *control nerves* and nodes’ density in sample areas in *stimulated nerves. NS* non-stimulated (control nerve), *S* stimulated nerve, *s* section, *a* sample area (n integer, number of nerve, section and sample respectively); **Down:** Node density of “non stimulated” and “stimulated” sample groups represented as a boxplot including average values and standard deviation.

The density—number of nodes per surface unit—was calculated for each sample area. The mean value of density calculated from the stimulated sample areas turned out to be significantly higher than the mean value of the controls (Fig. 3; Table 1). The mean values are 5.5 (SD 4.8) for controls and 37.0 (SD 24.9) for stimulated nerves. To compare the two means, we performed a simple two-tailed Student’s t-test and a Welch one way ANOVA, resulting in a difference statistically significant at a level *p* < 0.01 and *p* = 0.02, respectively.

**Table 1:** Nodes’ density in sample areas in control nerves and nodes’ density in sample areas in stimulated nerves.

Sample	Nodes density values in sample areas of CONTROL NERVES—Nodes/mm^2^	Sample	Nodes density values in sample areas of STIMULATED NERVES—Nodes/mm^2^
NS1 a1	3.6	S1 a1	15.6
NS1 a2	3.5	S1 a2	55.5
NS1 a3	9.3	S2 s1 a1	67.1
NS1 a4	1.9	S2 s1 a2	124.6
NS1 a5	2.4	S2 s1 a3	32.5
NS2 s1 a1	4.1	S2 s1 a4	28.7
NS2 s1 a2	7.0	S2 s1 a5	54.3
NS2 s1 a3	10.8	S2 s1 a6	50.8
NS2 s1 a4	6.8	S2 s1 a7	41.0
NS2 s1 a5	8.8	S2 s1 a8	33.9
NS2 s1 a6	9.5	S2 s1 a9	37.7
NS3 s1 a1	26.3	S2 s1 a10	55.9
NS3 s1 a2	4.7	S2 s1 a11	19.2
NS3 s1 a3	9.5	S2 s1 a12	20.2
NS3 s1 a4	11.9	S2 s1 a13	29.4
NS3 s1 a5	5.0	S2 s1 a14	0.0
NS3 s1 a6	3.9	S2 s1 a15	21.4
NS4 a1	9.8	S2 s1 a16	42.2
NS4 a2	3.8	S2 s2 a17	33.9
NS4 a3	5.0	S2 s2 a1	38.2
NS4 a4	3.6	S2 s2 a2	72.5
NS4 a5	2.9	S2 s2 a3	37.9
NS5 a1	1.1	S2 s2 a4	29.3
NS5 a2	0.0	S2 s2 a5	67.0
NS5 a3	0.0	S2 s2 a6	75.0
NS5 a4	0.0	S2 s2 a7	82.2
NS5 a5	0.0	S2 s2 a8	27.5
NS5 a6	0.0	S2 s2 a9	22.4
NS5 a7	0.0	S2 s2 a10	52.2
NS6 a1	6.1	S2 s2 a11	11.6
NS6 a2	0.3	S2 s2 a12	13.8
NS6 a3	0.0	S2 s2 a13	13.5
NS6 a4	9.8	S2 s2 a14	20.6
NS7 s1 a1	5.3	S2 s2 a15	0.0
NS7 s1 a2	5.3	S2 s2 a16	40.2
NS7 s1 a3	9.7	S3 s1 a1	29.3
NS7 s1 a4	9.7	S3 s1 a2	61.7
NS7 s2 a1	6.7	S3 s1 a3	30.9
NS7 s2 a2	8.7	S3 s2 a1	38.9
NS7 s2 a3	8.8	S3 s2 a2	92.5
NS7 s2 a4	4.8	S3 s2 a3	48.9
NS8 a1	4.0	S4 s1 a1	9.5
NS8 a2	1.9	S4 s1 a2	11.4
		S4 s2 a1	8.9
		S5 s1 a1	34.0
		S5 s1 a2	22.2
		S5 s2 a1	23.4
		S5 s2 a2	12.6
		S6 a1	48.6
		S6 a2	8.6

The values reported below correspond in detail to the Fig. 3 data.

*NS* control nerve, *S* stimulated nerve, *s* section, *a* sample area.

**Figure 4:**
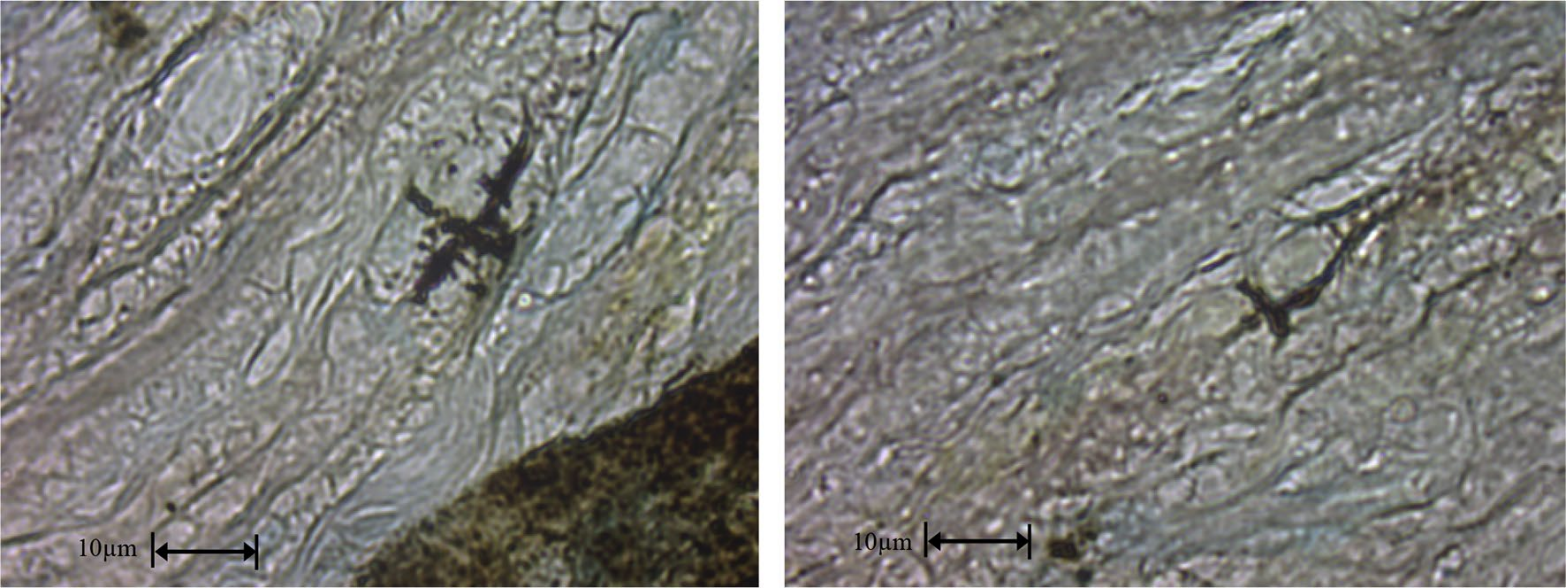
Two different examples (left and right) of Ag deposition (black) in the paranode after electrical stimulation of the rat sciatic nerves.

A significant difference between cases and controls is shown, demonstrating the occurrence of the phenomenon in association with nerve activity. Some findings, though limited in number, are worth to be mentioned, for their possible significance, which will be considered in the discussion section. In some specimens a spiral arrangement of Ag impregnation, as shown in Fig. 4, was observed in the paranodal region, resembling typical canalicular structures of noncompacted myelin, as described in histologic studies^20^.

## Discussion

### Photographic effect in the node of Ranvier after electric stimulation

Our study demonstrated experimentally the occurrence of a significant photographic effect in the node of Ranvier after electric nerve stimulation, in total absence of external or artificial light sources.

Our findings have come in a field of research that is attracting growing interest, namely the electromagnetic aspects of action potential and neural communication^2,21^. The topic of light transfer through nervous tissues has been addressed by previous studies, either experimentally or by modeling^12,13^. Beyond the evidence of the role of photons in different forms of cellular communication^22^, the hypothesis that light may play some role in neurotransmission has been corroborated by the possibility that the myelin sheath behaves like a waveguide, whereas the origin of photons is controversial^8,13^.

However, if myelin works as a waveguide, it remains to be explained what happens in the nodal gaps, where the myelin sheath is interrupted. Our group proposed the hypothesis that photons originate in the node of Ranvier and described a model of electromagnetic radiation emitted from nodal ionic currents^19^. By introducing in the simulation known dimensional parameters and physical properties of the involved biological structures (i.e. node, paranode, myelin, sodium channels), the patterns of the electromagnetic fields and wave propagation in the node-myelin complex resulted to lie in the visible and infrared bandwidth. Since this interval includes wavelengths suitable for Ag^+^ photoreduction, the present findings are consistent with the proposed model of photonic radiation in the node of Ranvier. An interesting issue concerns whether wavelengths not able to reduce Ag^+^ participate in the overall process. In a recent study, the possibility of THz wave propagation in the myelin was investigated by simulation^15^. We cannot exclude other phenomena and electromagnetic radiation at different wavelengths. Indeed, the method of Ag^+^ photo-reduction is most effectively able to detect photons in the visible range which is a portion of wavelengths provided by our theoretical model.

Even though the aim of the study is to investigate the photographic effect in the node, the incidental finding of silver granules in the paranodal domain is worth to be discussed.

Kumar et al. argued that a possible issue of future research would be to demonstrate experimentally the presence of photons in axonal structures, suggesting that Ag^+^ in solution injected in the cytoplasmic loops could precipitate in Ag granules when exposed to photons^13^. Interestingly, our findings showed some Ag depositions visible in the paranode, in correspondence of the typical spiral shaped structures of non-compacted myelin described in anatomical studies^20^. These structures communicate with the extracellular medium and can be perfused by Ag^+^ in solution, which precipitates as Ag when exposed to radiation.

In fact, this site is located right in the trajectory of photons, as predicted by the described model^19^. Nevertheless, Ag granules were sparse and, similarly, no significantly representative Ag deposition was visible in the axon lumen, as was the case in Sun’s study^12^. This could be explained by the fact that nodes are more exposed to solutes, while a longer bathing time would be needed to obtain an efficient perfusion of the paranodal structures.

### Photonic radiation detected

In this study, we have demonstrated the presence of photonic radiation in the node of Ranvier by means of a photographic technique. These results open new perspectives towards the hypothesis that photons may play a role in the transfer of information from one node to another or even contribute to the action potential. In relation to this, further considerations can be made. The model of electromagnetic radiation coming from the node we recently proposed^19^ and that one concerning the optical transmission through a myelin waveguide, previously described by Kumar and other authors^13–16,19^, are consistent with each other and with our experimental results and can be considered as a more comprehensive model, describing the whole sequence of generation and transmission of photons, in the node and through the myelin respectively. However, hypotheses describing how photons act when they reach the next node are still missing and deserve further research^13,19^.

### Possible explanations for the photonic activity: what is the effect of photons in the next node?

A possible effect to investigate is the influence on channel gating. Ion channel stimulation by light has been subject of many studies. For example, with the purpose of functional analyses of neural circuits, a method for optical stimulation of genetically designated neurons was developed^23^. Although this method and the following widespread use of similar techniques allowed a variety of studies on the physiology of neural circuits by optical stimulation, photosensitivity was genetically induced and a possible physiological role of photons in neural function remained unclear. The main issue of our study and previous works on the same topic is to investigate whether light sensitivity is an intrinsic property of the neural system and play a more extensive role in neurotransmission, other than retinal photoreception. Light responsive ion channels are widely represented in nature, throughout the history of cellular evolution, from prokaryotes to retinal cells^24^. Although light sensitivity has become predominant during evolution in some channels due to their special function, many other channels, beside voltage sensitivity as the main mechanism of operation, show some response to light^25,26^. Furthermore, the reactivity of non-photosensitive neurons to infrared laser radiation has been attributed to TRPV4 ion channels due to thermal activation, although the exact mechanism is unclear^27–29^.

Another possible role of photons in node to node signaling may involve ion permeation. For example, they could influence the energetic state of ions in the channel or at molecular binding sites^30^. Further studies are needed to gain a more comprehensive understanding of the mechanisms underlying the sensitivity to light of the molecular systems involved in neurotransmission, including channel proteins. In particular, we believe that it is worth to further explore the possible role of photons in ion channel operation.

Another interesting issue that arises is the possible relevance of some quantum effects in neural communication, which has been the subject of numerous studies in recent decades, providing an alternative to the purely classical approach to some phenomena that are still unexplained.

In fact, the so-called quantum biology has been the subject of much literature and experimental studies, from the cellular level to the highest brain functions, including an intriguing, though controversial, interpretation of consciousness^31–35^.

The proof that photons occur in the node of Ranvier and in other structures of myelinic axon, suggests that they may actively participate in the processes of neurotransmission. Nonetheless, it offers novel arguments of investigation by a quantum mechanical approach, beside classical electromagnetism, in neuroscience. In fact, Ag^+^-photoreduction method is most effectively able to detect exposure to photons, but it does not allow an evaluation of their statistical properties. Thus, the results of the present study can be considered as the starting point for further investigation, exploring the phenomenon from a quantum mechanical point of view. To achieve the goal of studying the statistical properties of the field, we think that a measuring device, capable of detecting very weak sources, is needed. In literature, an interesting method for photon emission detection which makes use of superconducting nanowire single-photon detector (SNSPD) is described. In particular, this can be found in a recently published work^36^ from Chenglong You et al., which seems a very interesting approach, exploiting the advantages of self-learning features of artificial neural networks and the naive Bayes classifier in the photon emission detection and offering new possibilities in terms of studying extremely weak light sources.

Some considerations, pertaining to Health topics, deserve further discussion.

In the last decade, laser stimulation techniques have reached a significant development, both in research and in clinical application, showing beneficial effects in many medical conditions such as chronic pain, chronic inflammation and even in nerve repair^26,37^.

A very interesting research topic is the influence of laser light in the visible wavelength range on nervous tissue regeneration. In particular, neurite growth has been observed in rat primary cortical neurons after 710 nm light irradiation^38^ and numerous studies have assessed sciatic nerve regeneration after injury, following treatment with visible radiation of different wavelengths^39,40^.

Concerning medical applications, laser therapy, especially in the form of low-level-laser therapy, a form of light therapy using wavelengths of 600–1000 nm, showed to be effective in different medical conditions, many of which pertaining to the nervous system^41,42^.

This has stimulated a growing interest in the mechanisms underlying nervous response to light. For example, Liebert et al. have hypothesized the existence of a mechanism of integrated and rapid modulation of ion channels, facilitated by photons, which would influence the transmission of neural information both in the peripheral and in the central nervous system^43,44^.

In relation to all the previous discussion points, the presence of photons in the activated axon suggests the possibility that the light-sensitive properties of neurons are intrinsic rather than an aspecific responsiveness to the electromagnetic stimulus. A deeper knowledge of the role of photons in neurotransmission and neuromodulation would contribute enormously to further development of the already promising therapeutic potential of laser light in neurology.

For the same reasons illustrated above, it cannot be excluded that photons play a role in regulatory networks of proteins involved in the homeostasis of the node-myelin complex. This may be considered as a functional unit, whose anatomical and ultrastructural characteristics are subject to fine tuning to regulate the conduction features^45^. Such a dynamic balance is maintained by the interaction of glial cells with ion channels, other membrane proteins and the cytoskeleton^46^. Impairment of this complex is often associated to the development of neurodegenerative diseases, such as multiple sclerosis^47^. Therefore, further studies on the possible role of photons in the regulatory mechanisms of node-myelin functional unit may provide a better knowledge and new therapeutic options for neurodegenerative diseases.

## Methods

### General procedure

The experiments were carried out on sciatic nerve segments from previously euthanized adult Wistar rats (400–500 g) at the Centre for Experimental Research, Università Cattolica del Sacro Cuore, Roma, Italy. Euthanasia was carried out according to the National Health Service *Guide for the Care and Use of Laboratory Animals* and it has been approved by the Institutional Animal Care and Use Committee at Università Cattolica del Sacro Cuore and Italian Ministry of Health (Project No. 1F295.N.WD5).

Aim of the procedure was to expose the nerve segment to Ag^+^ in solution, during electrical stimulation, in order to ascertain if Ag granules, as a result of photo-activated Ag reduction, could be detected specifically in the nodes by microscopy.

The principle by which photons should be detected is photoreduction of ionic Ag^+^ (colorless) to Ag, which precipitates as insoluble black granules^48^. Based on this principle, a method of “in situ biophoton autography” (IBA) has been described by Sun et al. This method is a staining technique with a silver ionic compound, AgNO_3_. The Ag precipitation mechanism here exploited, is the same as in photography, i.e. reduction of Ag^+^ to Ag by light exposure and subsequently Ag deposition in situ. Deposited dark Ag granules are insoluble in water and organic solvents such as alcohol and xylene, and they can be identified morphologically under common light microscopes. Ag^+^ was introduced in the form of an aqueous 10% solution of AgNO_3_ during nerve stimulation and subsequently removed by means of sodium thiosulphate (Na_2_S_2_O_3_) to remove all the residual not reduced silver cations, thus avoiding further subsequent precipitation due to environmental light exposure after withdrawal of the specimen. In addition, the thiosulphate treatment allows to complex silver cations that could be precipitated eventually as silver oxide (dark brown in color), that can be misinterpreted as solid Ag. The stoichiometry of the reaction is 1:2 in thiosulphate, to avoid the known formation of Ag_2_S and promoting the known complexation reaction described above:

1 $${\text{Ag}}^{ + } + {\text{ 2 S}}_{{2}} {\text{O}}_{{3}}^{{{2} - }} \to \, \left[ {{\text{Ag}}\left( {{\text{S}}_{{2}} {\text{O}}_{{3}} } \right)_{{2}} } \right]^{{{3} - }}$$

Stimulation of neural tissues was obtained by light emitting diodes, in a completely darkened container designed to avoid light exposure of the immersed tissues. In these conditions, the silver cations reduction could only be ascribed to the light transmission through the tissue^12^.

Similarly, our experimental procedure was designed to avoid exposure to environmental light during nerve stimulation. To this purpose all the nerve treatment steps, reported in the following description, were carried out in a dark polystyrene isothermal box, to obtain total insulation from external light.

The steps for chemical treatment of the nerve were as follows: (1) tissues immersed in a 10% AgNO_3_ solution in a container for 30 min in a dark box in a dark room; (2) rinse in 252 mM sucrose for 15 min; (3) fixation by 10% formaldehyde for 5 min; (4) rinse in de-ionized water for 5 min; (5) rinse in 10% sodium thiosulfate for 15 min; (6) fixation by 10% polyformaldehyde. Steps 1–5 followed the technique described by Sun et al^12^.

As in IBA, stimulation was applied during step 1. However, in order to avoid the use of any artificial light source, in our study compound action potentials were elicited electrically, by means of a square wave electric stimulus having the following characteristics: voltage magnitude = 3Vpp, frequency = 20 Hz (i.e., 50 ms of time period), duty cycle = 5% i.e. on duration about 2.5 ms, off time 47.5 ms.

The experimental setting was prepared as follows: a nerve stimulation chamber (*Nerve chamber, BIOPAC Systems, Inc.*) was placed in the dark box. The tube for infusion of bathing solutions and electric wires were inserted through holes into the box and protected from external light by drapes and envelopes. After positioning the electrodes and connecting the infusion tube to the chamber, the volume to be injected was previously calibrated at the adequate level to avoid contact with the stimulation grid during the procedure.

After euthanasia, sciatic nerves were harvested by delicate dissection from crural to tibial region. Short segments were cut apart from each nerve to serve as controls.

**Figure 5:**
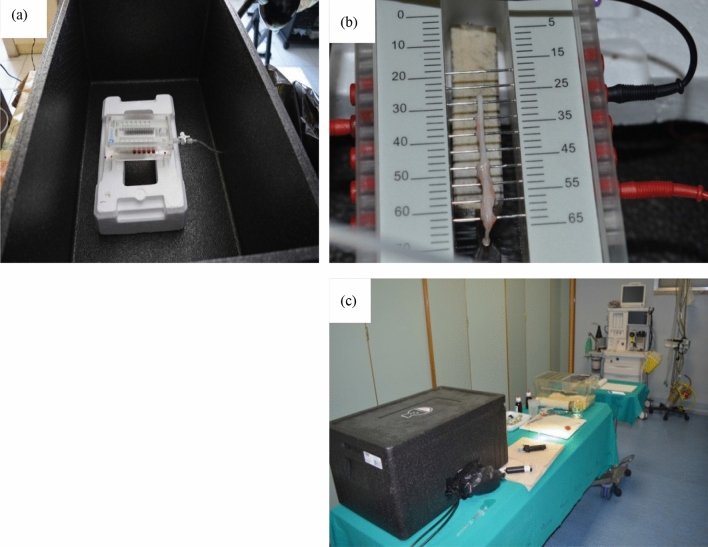
Experimental setup for nerve stimulation and chemical processing. (**a**) The Biopac nerve chamber in acrylic (*Biopac ®*); it features 15 stainless steel pins; each pin is spaced 5 mm apart to provide a variety of recording and stimulation configurations; the sockets accept 2 mm plugs and interface with stimulation and recording cables; the nerve chamber is secured within the polystyrene dark box to prevent environmental light from hitting the nerve. (**b**) Stimulation and measurement electrodes connected to the steel pins and a rat nerve placed on the pins. (**c**) The final lab setup: by means of a tube the different solutions for the chemical treatment of nerves can be introduced or extracted without opening the box; wires from the electrodes and the tube are passed through holes in the box wall and isolated by envelopes; the dark box is closed and ready for the experiment.

The prepared nerve was immediately placed on the stimulation grid of the nerve bathing chamber (*Nerve chamber, BIOPAC Systems, Inc.*) at room temperature. The distal end of the nerve was left hanging down for 1 cm in the reservoir of the chamber, to allow immersion in bathing solutions during stimulation. The box was closed and the procedure performed from step 1 to 6 (Fig. 5).

Control segments underwent the same procedure, except stimulation.

After completion of the procedure the box was opened and the specimen (the nerve distal segment previously immersed in solutions) was cut and placed in formaldehyde 10% for histology. At this step all the silver from the solution should have been either precipitated or removed by the treatment. However, in order to prevent precipitation of unexpected residual silver after exposure to environmental light, harvesting was performed in a dark environment.

After 48 h fixation in formaldehyde, the specimen was processed for embedding in paraffin. 5 µm thick slices were obtained, stained by toluidine blue standard technique and cover slipped.

### Digital images acquisition and statistical analysis

Digital images were acquired from the slides by *NanoZoomer Digital Pathology System C9600 series, Hamamatsu Photonics K.K.*, for histologic study. Each digitized image of nerve sections was examined by an image viewing software, *NDP.view2, Hamamatsu Photonics K.K.*. Image areas containing tissues other than nerve fibers, artifacts and regions of poor optical quality where discarded. Sample areas of tissue, corresponding to fascicular groups of fibers, were identified and measured by means of the viewing software tools (*NDP.view2, Hamamatsu Photonics K.K.*).

Within each area, the nodes of Ranvier containing silver precipitates were identified, marked and counted. The density of marked nodes (nodes/mm^2^) was calculated for each sample area as shown in Fig. 3. To compare the means of densities coming from non-stimulated and stimulated nerves, we performed several statistical tests with the help of the R platform^49^. Strictly speaking, our data do not fulfill the assumptions of classical parametric tests, such as two-tailed Student’s t-test and one way ANOVA. The two groups have remarkably different standard deviations and the data appear to be drawn from somewhat long-tailed distributions. Here we summarize the results of some tests (assuming that they are sufficiently robust to non-normality): pairwise t-test adjusted for different sd (*p* = 1.7e−8), Welch corrected one-way ANOVA (*p* = 1.7e−8), Kruskal–Wallis (*p* = 3.1e−9), Wilcoxon with continuity correction (*p* = 3.2e−9).

### Preparation of the solutions

Silver nitrate solution was prepared dissolving 10 g of AgNO_3_ (Sigma Aldrich, ≥ 99.0%.) in 100 ml of pure water (corresponding to a concentration 0.6 M). The sodium thiosulphate solution was prepared dissolving 20 g of Na_2_S_2_O_3_ (Sigma Aldrich, > 99%) in 100 mL of pure water (corresponding to a concentration of 1.3 M); the molarity of the second solution was about double the first one, in order to be sure to have the silver cation: thiosulphate anion in the 1:2 ratio according to the requested stoichiometric coefficient ratio (see Eq. 1).

Sucrose (C_12_H_22_O_11_) (Sigma Aldrich, BioXtra, ≥ 99.5% (GC)) solution 0.252 mM was prepared dissolving 43,13 g in 500 mL of pure water.
